# Traditional meat preservation techniques and their modern applications

**DOI:** 10.1093/af/vfaf051

**Published:** 2025-12-18

**Authors:** Samuel C Watson

**Affiliations:** Department of Animal Science, The Pennsylvania State University, University Park, PA 16802

**Keywords:** Charcuterie, Hurdle effect, Salumi, Traditional meat processing

Implications

- Traditionally processed meat products like prosciutto and salami rely on sound preservation techniques that are thousands of years old and originate from ancient regional cultures that to this day maintain modern iterations.
- In the US, small and very small meat processors balance the need to be profitable with the desire to maintain slow production methods that can hinder business growth in order to create products that are authentic to their roots.
- Traditional meat products have been a vital part of the human diet for thousands of years and still have a key role to play in the modern culture of food and diet.

## History of Traditional Meat Processing

Food preservation practices date to the beginnings of ancient societies, and the prosperity of an ancient society was often associated with the development of food preservation knowledge and skills. Key among those skills was the ability to harvest salt from natural sources and using salt to extend the shelf life of food (Bloch, 1976). Some meat processing techniques that are still widely used today pay homage to ancient skills like the fire-side smoking of hunter-gatherers and the salting, drying, fermenting, and curing techniques from cultures across the globe. In modern times, traditional in the context of food preservation culture is defined as originating from distinct historic cultures that are linked to specific locations (Bertozzi, 1998), and traditional foods call back to the places where they were initially created. In Europe, there are hundreds, if not thousands, of unique meat products and processes that have existed throughout the history of small towns and larger regions. The traditional concept of “charcuterie” originates from French butchers who were renowned for their preserved meat products, most often pork, and were recognized for their focus on preservation of the whole animal with little waste (Ruhlman and Polcyn, 2013). In a similar way, “salumi” refers to a culture of classical preparations of products like prosciutto, pancetta, coppa, guanciale, and salami that have to this day maintained strong regional identity across what has only recently become the nation of Italy (Ruhlman and Polcyn, 2012). The availability of traditional meat products for sale in US grocery stores is owed to the importing of the ancient food cultures of Europe to the US with recipes that were either brought directly or that emulate products from Italian, French, Spanish, Polish, or German heritages.

## The Hurdle Effect and Traditional Product Safety

A hurdle is a preservative component of a production process or ingredient addition that has an antimicrobial effect (Leistner, 2000). Traditionally processed meat products rely heavily on the hurdle effect and the combination of multiple antimicrobial hurdles through common processing steps like salting, drying, curing with nitrite, and fermentation. Compared to products made with modern thermal processing, traditional products are unique, because the same level of safety as a high temperature cooking process can be achieved without the heat. The antimicrobial effect of the processing steps each contributes to the lethality of pathogens synergistically (Leistner, 2000). Understanding how salting, drying, curing with nitrite, and fermentation impact food safety metrics like water activity and pH is key to understanding why traditional products are safe to eat.

Water activity is a measurement of the vapor pressure of a food product as a ratio of the vapor pressure of pure water, and it is used to express the amount of water that is free and available for microbial growth in a product. As water activity decreases, the more difficult it becomes for bacteria to survive and grow. Bacterial pathogens like *Escherichia coli* and *Salmonella* typically are not able to grow in foods with a water activity of 0.95 or lower, whereas *Listeria monocytogenes* and *Staphylococcus aureus* have lower water activity limits of 0.92 and 0.86, respectively (Sperber, 1983). Most traditionally processed meat products have a water activity around 0.90 to 0.85, depending on the style of the product.

Adding salt (sodium chloride) has a major impact on water activity, and salt is an antimicrobial because it interferes with the ability of a bacterial cell to maintain its internal water pressure, called turgor pressure. To remain viable, bacteria require the outward turgor pressure to prevent membranes from collapsing, and cells are constantly reacting to environmental changes to ensure that internal water and ion concentrations are maintained at optimal conditions. When high concentrations of sodium chloride are added to raw meat during processing, bacteria present in the meat will lose internal water, resulting in a loss of turgor pressure that hinders the cell’s ability to grow or survive in the meat product (Beales, 2004).

Drying has been used in tandem with salting during the manufacturing of traditional meat products since their earliest inception, and these processes work synergistically from a quality and safety perspective. From a quality perspective, drying intensifies flavors and creates the textural changes that are associated with dried sausages and hams. For traditionally dried hams in particular, applying salt to the lean surfaces of the raw hams draws moisture out of the raw ham, accelerating the moisture loss necessary to create the final product. From a safety perspective, drying decreases water activity by further concentrating the salt that has been added to the product. As salt concentration in the product increases, so too increases the stress experienced by pathogenic bacteria caused by changes in osmotic pressure in the product (Tapia et al., 2020).

Fermentation in the context of meat processing refers to the consumption of sugars by non-pathogenic bacteria resulting in the generation of organic acids and other metabolites. The creation of lactic and other organic acids decreases the pH, causing stress on bacteria present in the product while usually creating a tangy flavor profile. Generally, US style meat products tend to have more acid and a stronger, more intense acidic flavor, whereas traditional products that hold to European styles often have less acid and a more mellow acidic flavor. The last century has seen a dramatic change in how meat products are fermented with the invention of commercial starter cultures. Artisans from medieval and older times relied on environmental bacteria passively entering initial batches of a product and would add a small portion of the fermented batch into the fresh meat of the subsequent batches. This process, called back slopping, was widely used until the 1950s when direct addition starter cultures were commercialized and made available to the food industry (Incze, 1998). Today, multiple unique strains of starter cultures are available, and they have made fermentation more consistent and reliable.

In the context of food safety, lactic acid created during fermentation is an excellent antimicrobial, because it typically causes the maximum amount of bacterial stress at pH values most common in fermented meats. The target pH for a fermented product, depending on the style of the product, ranges from approximately 4.5 to 5.3. In this range of pH values, organic acid molecules hold on to their hydrogen atoms and pass through the cell membranes of bacteria in the product. Inside the cell, the pH is near neutral, and the hydrogen atom is no longer bound to the rest of the acid molecule and is dissolved freely in the cytoplasm. This decreases the internal pH of the bacterial cytoplasm and forces the bacteria to pump the hydrogen out of its cytoplasm, spending energy to do so (Beales, 2004). Bacteria that are tolerant to acidic pH can better withstand this stressor, but the energy expended on maintaining neutral pH inside the cell leads to cellular exhaustion and ultimately causes bacterial cell death.

Nitrites are the final major group of antimicrobial hurdles associated with traditionally processed meat products. For the purposes of this article, products cured with nitrite rely on the presence of nitric oxide to create cured meat color and cured meat flavor. In some culinary circles, salted and dried meat products are called “cured” or “salt cured” even though the product does not contain compounds that result in nitric oxide being present. Products that are cured with nitrite are produced by adding compounds that contain nitrites, nitrates, or both. These compounds are often added directly as purified sodium salts (i.e., Sure Cure, Cure No.1, or Cure No. 2) or increasingly as alternative curing compounds derived from plant sources. Although the implementation of conventional and alternative curing compounds is not always the same from a processing point of view, the end products will ultimately contain nitrites and other derivatives, like nitric oxide, that are chemically active in the product. The nitrites and other derivatives have been reported to slow the growth of pathogenic bacteria, especially in products that are salted. Additionally, nitric oxide is responsible for preventing the germination of spore forming bacteria like *Clostridium botulinum* and *C. perfringens*, possibly through the ability of nitric oxide to cause damage to the external membrane of the spores (Majou and Christieans, 2018). Research has shown that meat products created with no added nitrite allow pathogens, especially *Listeria monocytogenes*, to grow more easily while the product is stored at refrigeration (Xi et al., 2011).

## Modern Validations of Traditional Products

A significant body of research conducted over the last thirty years has contributed both broad concepts and specific process validations that support the safe production of traditionally processed meat products, with a selection of those works highlighted here. First, the implementation of the degree-hours concept as a method of preventing toxin production by *Staphylococcus aureus* has been a major benefit in improving the safety of fermented products. This pathogen can grow and produce toxin at or above 60 °F (15 °C), and ensuring that it does not grow to levels where toxin is produced is crucial for ensuring that a fermented product is safe to consume (The American Meat Institute Foundation, 1997). Degree-hours is the number of hours above 60 °F (15 °C) that a fermentation process can take at a given temperature to reduce the pH of the product to 5.3 or lower. At this pH, *S. aureus* growth is controlled. The maximum time a fermentation can take to reach pH 5.3 can be calculated using the degree-hours concept, with fermentations at 100 °F (37 °C) or higher requiring less than 900 degree-hours, fermentations between 100 °F (37 °C) to 90 °F (32 °C) requiring less than 1,000 degree-hours, and fermentations less than 90 °F (32 °C) requiring less than 1,200 degree hours (The American Meat Institute Foundation, 1997). Overall, this concept has vastly improved the safety of fermented meat products by controlling *S. aureus.* The success of this concept is one likely cause, alongside the widespread use of commercial starter cultures, for the ending of US Department of Agriculture testing for staphylococcal enterotoxins in 2003 (US Department of Agriculture, 2023).

During the production of a fermented and dried pepperoni product inoculated with *E. coli* O157: H7, a thermal process of 53 °C (128 °F) held for 60 min was shown to cause adequate lethality when paired with the fermentation and drying parameters used in this process (Hinkens et al., 1996). For pepperoni that was fermented (36 °C, 97 °F) with no additional thermal lethality, the storage of the pepperoni slices at room temperature (21 °C, 70 °F) after fermentation and drying was shown to be a key step in the production process for achieving maximum reductions in the inoculated pathogen (Faith et al., 1997). After 60 days of storage, *E. coli* O157: H7 was not detected in pepperoni stored aerobically at 21 °C (70 °F) versus a recovered *E. coli* O157: H7 concentration of 4.40 and 3.71 log_10_ cfu/g after 60 and 90 days of aerobic storage at 4 °C (39 °F), respectively. A subsequent study using the same fermentation and drying parameters to manufacture the pepperoni showed a similar impact of the room temperature holding step after fermentation and drying on inoculated *Salmonella* populations (Ihnot et al., 1998). After 56 days of production, *Salmonella* concentrations in product held at 21°C (70 °F) were less than 0.5 log_10_ cfu/g, and concentrations in product held at 4 °C (39 °F) were approximately 2.5 log_10_ cfu/g.

More recently, the addition of an organic acid processing aid has been shown to be an effective addition to the hurdles of producing raw, ready-to-eat charcuterie. Birko Beefxide, an antimicrobial blend that includes lactic acid and citric acid, was used at 2.5% application on raw trim during the manufacture of a salted, cured, and dried beef bresaola. Reductions of at least 5 log_10_ were achieved in *E. coli* O157: H7, *Salmonella*, and *Listeria monocytogenes* populations by the end of the complete manufacturing process (Watson et al., 2021). Beefxide was also used for treatment of raw trim in the manufacture of non-thermally processed pork salami (McKinney et al., 2019). The manufacturing processes were able to achieve reductions of at least 5 log_10_ in *Salmonella* and *Listeria monocytogenes* populations in both products. One challenge that was highlighted in the manufacture of the pork salami was the difficulty in controlling *E. coli* O157: H7. While this pathogen has not been historically associated with the colonization of pigs, using natural beef casings for larger diameter salami products is a common practice, therefore establishing the need to evaluate pathogenic *E. coli* for this product. The study found that *E. coli* O157: H7 populations in pork salamis treated with Beefxide and stuffed into natural beef casings were only reduced by 3.92 log_10_. Processors making pork salami products without the use of a thermal lethality step are recommended to verify that concentrations of *E. coli* in the raw meat and casings are less than 2 log_10_. Overall, this highlights the need for processors to fully understand the nuances of their process along with verifying that they are correctly following the critical parameters established in the validation work.

## A Modern Processor’s Perspective on Traditional Processing

Tony Incontro, the founder and operator of Incontro Cured, a ready-to-eat meat processing business in Lincoln, NE, was asked to share his artisan perspective on traditional products, the challenges faced by this segment of the meat industry, and what he thinks the future of traditional processing will look like. With his family, Incontro also raises Mangalitsa pigs, a highly marbled heritage pork breed that has been brought to the US from Hungary in the last twenty years. Together with other Mangalitsa producers in the US, Incontro produces traditional salumi products with this pork breed. On his definition of traditionally processed meats, Incontro stated: “Traditionally processed meats are a testament to culinary heritage—carefully crafted using age-old techniques that honor both flavor and preservation… the process is slow, intentional, and rooted in place. Whether it’s a delicately aged prosciutto from Italy, a boldly spiced chorizo from Spain, or a rustic sausage from Eastern Europe, each product carries a story of land, tradition, and generations of hands that shaped it.”

Incontro acknowledged that inherent to the production of traditionally processed meats are some unique challenges. “Because these products rely on time-intensive methods like curing, aging, and natural fermentation, scaling up production is difficult—it’s hard to maintain consistent quality and flavor when increasing very large volumes,” he said. “Additionally, sourcing high-quality, pasture-raised animals in sufficient quantities can be a challenge, especially when working with small, local farms. Seasonal variations, limited supply, and the need for sustainable practices often limit how much product can be made at once.”

Incontro also shared his perspective on the challenges associated with producing traditional meats under federal oversight. He said, “Another major challenge is navigating the strict USDA guidelines and food safety regulations designed to protect consumers. While these rules are essential, they can be complex and costly for small-scale, artisanal producers to comply with—especially when traditional, artisan methods don’t always fit neatly into modern regulatory frameworks. Balancing compliance without compromising authenticity and craftsmanship requires careful planning and sometimes significant investment.” In 2023, USDA-FSIS published the “FSIS Ready-to-Eat Fermented, Salt-Cured, and Dried Products Guideline” with the intent of highlighting the agency’s current thinking on meat products that do not rely on cooking as the primary lethality step. While not exclusionary of large processors, the purpose of this guidance was to support the small and very small meat processors by providing technical assistance that Incontro and many others in the traditional products sector seek.

Lastly, Incontro gave his perspective on how he sees the traditionally processed products sector changing in the future. “The [traditional products] industry will need to balance scalability with craftsmanship—finding ways to expand production without sacrificing the patience, quality, and heritage that define these products. Advances in technology may help monitor and control aging environments or improve food safety while still respecting traditional methods,” he said. Leaning on his experience raising Mangalitsa pigs and working with local producers, he added, “We can also expect stronger partnerships with small, local, and heritage farms to ensure a steady supply of superior raw materials that prioritize animal welfare and environmental stewardship. This could spur a renaissance of regenerative agriculture and revive heirloom breeds once common in regional diets.” He closed by saying, “Education and storytelling will play a key role in connecting consumers with the cultural and nutritional value of these meats—helping them appreciate the craft and care behind every bite… The next 50 years could see the traditionally processed meat industry thrive as a bridge between past and future—preserving age-old traditions while embracing innovation and sustainability to meet evolving tastes and values.”

## Conclusions

The traditional meat processing methods of salting, drying, fermenting, and curing have been a staple of the human diet for millennia, and they are capable of controlling the foodborne bacteria pathogens that are inherent to the environments where the animals are raised and the products manufactured. Traditional products are also a labor of love, and being successful in this sector requires an understanding that it will take longer to see the fruits of those labors than other parts of the industry. Small producers and artisans are optimistic about the future of their products, and they should not be overlooked. In light of global policy debates on what should be allowed in the diet and financial incentives that ruthlessly pursue production speed and the highest possible margins, we should not lose sight of the societal value traditional meat products provide. These products are worth their effort, and it would be an incredible tragedy to allow them to be lost to convenience.

## Author Biography

**Sam Watson** is an Assistant Professor of Meat Science in the Department of Animal Science at The Pennsylvania State University. He obtained a Ph.D. from the University of Nebraska—Lincoln in Meat Science. His research and extension activities are focused on improving the safety of meat products and the meat processing environment and on providing training to the meat industry.

Corresponding author: scw186@psu.edu
